# Does Wearable-Measured Heart Rate Variability During Sleep Predict Perceived Morning Mental and Physical Fitness?

**DOI:** 10.1007/s10484-022-09578-8

**Published:** 2023-01-09

**Authors:** Herman de Vries, Hilbrand Oldenhuis, Cees van der Schans, Robbert Sanderman, Wim Kamphuis

**Affiliations:** 1 Research Group Digital Transformation, Hanze University of Applied Sciences, Groningen, The Netherlands; 2 Department of Human Behaviour & Training, Netherlands Organization for Applied Scientific Research (TNO), Kampweg 55, 9769 DE Soesterberg, The Netherlands; 3 Department of Health Psychology, University Medical Center Groningen, Groningen, The Netherlands; 4 Department of Rehabilitation Medicine, University Medical Center Groningen, Groningen, The Netherlands; 5 Research Group Healthy Ageing Allied Health Care and Nursing, Hanze University of Applied Sciences, Groningen, The Netherlands; 6 Department of Psychology, Health and Technology, University of Twente, Enschede, The Netherlands

**Keywords:** Heart rate variability, Sleep, Resilience, Ecological momentary assessment, Wearables, Military

## Abstract

The emergence of wearable sensor technology may provide opportunities for automated measurement of psychophysiological markers of mental and physical fitness, which can be used for personalized feedback. This study explores to what extent within-subject changes in resting heart rate variability (HRV) during sleep predict the perceived mental and physical fitness of military personnel on the subsequent morning. Participants wore a Garmin wrist-worn wearable and filled in a short morning questionnaire on their perceived mental and physical fitness during a period of up to 46 days. A custom-built smartphone app was used to directly retrieve heart rate and accelerometer data from the wearable, on which open-source algorithms for sleep detection and artefact filtering were applied. A sample of 571 complete observations in 63 participants were analyzed using linear mixed models. Resting HRV during sleep was a small predictor of perceived physical fitness (marginal R^2^ = .031), but not of mental fitness. The items on perceived mental and physical fitness were strongly correlated (r = .77). Based on the current findings, resting HRV during sleep appears to be more related to the physical component of perceived fitness than its mental component. Recommendations for future studies include improvements in the measurement of sleep and resting HRV, as well as further investigation of the potential impact of resting HRV as a buffer on stress-related outcomes.

**Supplementary Information:**

The online version contains supplementary material available at 10.1007/s10484-022-09578-8.

## Introduction

Occupational stress can lead to physical (Kivimäki & Kawachi, 2015; Yang et al., 2019) and mental (Chirico, 2016) health problems, decrease quality of life (Bhattacharya & Ray, 2021) and imposes a financial burden on society via absenteeism and productivity loss (Hassard et al., 2018). Early recognition of the potential development of stress-related problems can be useful for personalized just-in-time interventions that may help alleviate or prevent these personal and societal burdens of stress (Wang & Miller, 2020). Due to recent developments in wearable sensor technology, continuous and unobtrusive measurement of physiological and behavioral data that may be related to stress resilience, is becoming increasingly feasible (De Vries et al., 2019; Drury et al., 2019). One of the challenges for current research on this topic is to explore and verify to what extent these novel sources of personal data can indeed be related to one’s ability to resiliently cope with stress.

Before hypothesizing how wearable-measured data may be related to resilience, it is important to understand how stress itself emerges. Stress is the outcome of a psychological process that is known as appraisal (Lazarus & Folkman, 1987). When a person is faced with demands, the brain subconsciously assesses the perceived availability of resources to cope with the situation. When sufficient resources appear to be available, the demand is appraised as a challenge. When this is not the case, the demand is appraised as a threat—causing a stress response. Therefore, the subjective assessment of the availability of resources is what determines the stress response. This can be measured as the perceived fitness, which is defined as “the modifiable capacity to utilize resources and skills to flexibly adapt to challenges or advantages” (Robinson et al., 2015). Since appraisal is a psychological process, it is not the person’s objective fitness-related characteristics that are directly assessed during appraisal, but the person’s *perceived* fitness. For instance, an objectively fit but insecure person may experience stress when confronted with a minor challenge that the person should easily be able to handle. Unfortunately, it is currently not possible to directly measure mental states like perceived fitness in an automated and unobtrusive way. However, if relevant physiological or behavioral data from wearables can be linked to it, it may be possible to use these measures as a proxy for perceived fitness in future studies and applications.

One metric that may be related to perceived fitness is Heart Rate Variability (HRV). HRV is a measure for the variation in the inter-beat-intervals (IBIs) between heartbeats that functions as a proxy for autonomous nervous system functioning (Thayer et al., 2012). Throughout the day, HRV is continuously influenced by factors such as stress (Kim et al., 2018) and emotions (McCraty et al., 1995), body posture (Buchheit et al., 2009), exercise (Michael et al., 2017) and intake of caffeine (Koenig et al., 2013) or alcohol (Romanowicz et al., 2011). HRV measurements are therefore context-dependent and fluctuate throughout the day, but when measured in a similar resting state where confounders are minimized (e.g., during sleep or upon awakening), accurate measurement of resting HRV is possible, even with consumer-available wearables or the camera of a smartphone (Plews et al., 2017; Stone et al., 2021).

Resting HRV has been consistently linked to diverse aspects of mental functioning. For instance, prior studies found that on a between-subject level, resting HRV is positively associated with cognitive flexibility (Colzato et al., 2018), affective flexibility (Grol & De Raedt, 2020), emotion regulation (Holzman & Bridgett, 2017; Mather & Thayer, 2018) and resilience (An et al., 2020). Two recent studies also found that on a within-subject level, resting HRV buffered the positive associations between stress and negative affect (da Estrela et al., 2021), as well as between stress and both demands and mental exhaustion (de Vries et al., 2021). These findings indicate that having a high resting HRV generally reflects more optimal mental functioning and adaptability to environmental demands, which makes it a potential proxy for perceived fitness.

Besides being linked to these mental aspects that may be related to perceived fitness, resting HRV has shown to be associated with physical components of fitness as well. On a between-subject level, resting HRV is positively associated with cardiovascular fitness (Souza et al., 2021; Tomes et al., 2020), and negatively associated with overuse injuries (Gisselman et al., 2016; Lima-Borges et al., 2018; Williams et al., 2017) and pain perception (Forte et al., 2022). Finally, resting HRV has also been linked to viral infections on a within-subject level (Conroy et al., 2022). These associations are the basis for HRV guided training, in which daily resting HRV is being used in comparison to the personal baselines of athletes to determine their physiological recovery from prior physical or mental stress and adjust training plans when necessary (Düking et al., 2021; Manresa-Rocamora et al., 2021). In this setting, the objective resting HRV data are often combined with subjective questionnaire data in order to get a more complete view of the athlete’s current status. Since resting HRV has been linked to both mental and physical aspects of fitness, it is possible that its potential association with perceived fitness may also differ for the perceived mental and physical fitness.

Wearable-measured resting HRV has been linked to diverse aspects of mental and physical functioning. As such, it may also be linked to a person’s overall perception of fitness. From the perspective of appraisal theory, this is relevant, since a person’s overall perception of fitness can be considered a resource to deal with demands. When this resource is perceived to be lacking, the person may be more susceptible to experience demands as stressors, and develop more stress-related complaints as a results. Exploration of the degree in which within-subject differences in resting HRV are indeed associated with perceived fitness will benefit the current state of knowledge on how HRV relates to subjective mental and physical functioning. Furthermore, insights in this association may be useful for the development of tools that provide automated and personalized feedback on its users’ readiness to handle demands and cope with stress. Such tools may be useful in intervention programs that aim to prevent stress-related problems. These insights are therefore particularly relevant for high-risk professions such as military personnel, in which resting HRV has already been related to objective fitness and occupational performance (Tomes et al., 2020). Therefore, this study aims to explore to what extent wearable-measured resting HRV during sleep predicts the perceived mental and physical fitness of military personnel on the subsequent morning. We hypothesize that wearable-measured resting HRV during sleep predicts both the mental and physical aspects of perceived fitness on the subsequent morning.

## Methods

An observational study was performed based on within-subject nested daily observations. The study protocol was approved (case 2019-038) by the internal Research Ethics Committee of TNO (TC-nWMO) in the Netherlands. The Strengthening the Reporting of Observational Studies in Epidemiology (STROBE) statement was used as a guideline for reporting (Von Elm et al., 2007).

### Participants

A convenience sample of 73 employees of the Dutch military were recruited to participate and collect data for a period of up to 8 weeks. This group consisted of 43 marines in training and 30 staff members of the Dutch Defense Healthcare Organization. Both the recruitment and data collection of this study was performed in the summer of 2019 at peacetime, in the Netherlands. Recruitment was facilitated by the Dutch military, but participation occurred on a voluntary basis and participants were free to stop at any time without adverse consequences. All participants gave explicit consent for the use of their (health) data.

### Data Collection

Descriptive data such as the age, gender and function of the participants were not collected out of privacy and security concerns related to the sensitive profession of the participants. Out of privacy and security concerns related to the military context of this study, it was not deemed acceptable to store the participants’ data on servers outside the jurisdiction of the Dutch government, which would have been the case during regular use of the Garmin wearables. As such, descriptive statistics could only be provided based on the daily measurements of the independent and dependent variables, and no subgroup analyses were performed.

#### Independent Variable: Heart Rate Variability During Sleep

All participants wore a Garmin Tactix Charlie smartwatch which is described as a multisport GPS watch with additional tactical functionality (Garmin & subsidiaries, n.d.). Therefore, a custom-built smartphone application was used that utilized the Garmin Health Standard Software Development Kit (SDK), which allows the application to collect data directly from the wearable device and process and store it on a self-hosted server (Garmin, 2022). Using this approach, data on accelerometry and green-light photoplethysmography-based IBIs between heartbeats were available, based on which sleep episodes and the related resting HRV can be detected and calculated.

Sleep detection was performed based on an open-source algorithm that detects sleep based on wrist movements (Hees et al., 2015), with three adjustments. First, the parameter that describes how long the user must lie still before that period is classified as ‘in bed’ was lowered from 30 to 10 min. This was done because pilot tests of the applied algorithm showed that the original algorithm sometimes classified a full night sleep as separate sleep episodes when a participant was awake at night, which can be prevented by lowering this threshold. The second adjustment was done with the same goal, by adding a parameter that allowed participants to have a period of up to 10 min of small movements during (restless) sleep, without being classified as awake and thus potentially splitting the sleep episode. Finally, an adjustment was made in how the start of the sleep episode was detected. Initially, the start of a sleep episode was estimated based on accelerometer data, as per the original algorithm. The start of the sleep episode was then adjusted to use the timestamp of the peak in the HRV during the first 30 min of that episode (based on the 90 s time window with the highest HRV) was then attributed as the actual start of the sleep episode. This was done because pilot tests showed that the original algorithm sometimes classified a period during which participants were lying still but not sleeping (e.g., reading on smartphone) as sleep, and prior research showed that HRV briefly peaks around the start of the sleep episode (Boudreau et al., 2013). Since this study compares the perceived mental and physical fitness of the participants during the morning to their resting physiology, the nocturnal HRV data was then related to the subsequent morning’s Ecological Momentary Assessment (EMA) questionnaire during statistical analysis. Finally, the Total Sleep Time (TST; the total duration of the sleep episode spent asleep) in hours and Resting Heart Rate (RHR; the average heart rate during sleep) were included as control variables.

The HRV was then calculated for each sleep episode. Since motion artefacts are common in real-life wearable-based measurements and can influence the accuracy of the HRV estimation, an artefact detection algorithm that has been used in prior research was used (Plews et al., 2017). This method consists of two steps. First, intervals are removed when they differ more than 75% from the previous one. Second, outliers are removed by including only intervals that are within less than 25% of the first quartile and within more than 25% of the third quartile. Additionally, sleep episodes where valid IBIs were available for less than 64% of the duration of the sleep episodes were discarded. This was done because prior research has shown that the rMSSD can be validly determined without clinically significant change (a 5% change in mean absolute percent difference) when up to 36% of the IBIs are removed (Sheridan et al., 2020). This study also showed that frequency domain HRV parameters are much more impacted by missing data and thus less robust in this context than time domain parameters. Another study confirmed that of all time and frequency domain HRV measures, rMSSD is one of the two (alongside mean NN) most robust features (Baek & Shin, 2017). Therefore, the root Mean Square of the Successive Differences (rMSSD) in milliseconds was used as the primary HRV variable and calculated based on the valid IBIs of the respective sleep episodes.^1^ This metric was then logarithmically transformed (lnrMSSD) to improve its distribution for statistical modeling, which is a common procedure in HRV research (Shaffer & Ginsberg, 2017).

#### Dependent Variables: Perceived Mental and Physical Fitness

Participants filled in a brief EMA questionnaire in the morning that included two items on their perceived mental and physical fitness, each of which scored on a 11-point Numeric Rating Scale (NRS) ranging from 0 to 10. Perceived physical fitness was assessed based on the item “I feel physically fit”, whereas perceived mental fitness was inquired via the item “I feel mentally fit”. These items were originally self-composed, but align well with items of the Acute Readiness Monitoring Scale (items 5 and 13) that has since then been validated for the use in military personnel (Keegan et al., 2021). Finally, the participants already were used to distinguish between mental and physical fitness based on their professional training and functioning. For these participants, perceived physical fitness is about feeling physically ready to perform (e.g., strength, endurance, mobility), whereas perceived mental fitness is related to feeling mentally (e.g., cognitively and emotionally) ready to perform.

### Data Analyses

All data-management and analyses were performed in RStudio (RStudio Team, 2020) and R (R Core Team, 2020). Descriptive statistics on the HRV, TST, as well as the perceived mental and physical fitness of the participants were calculated. Due to the difference in scales between HRV, TST and the EMA items, standardizing the data was necessary to optimize the comparability of the coefficients of the independent variables. Standardization based on the within-subject values was considered since the level 1 association between HRV and the EMA items is of primary interest (Enders & Tofighi, 2007), but standardization at the grand mean was finally preferred, as some participants collected a relatively low number of complete observations.

Two two-step hierarchical linear mixed-effects models for each of the EMA outcomes were created using the “lme4” package in R (Bates et al., 2015) to account for repeated measures within participants. All models were based on fixed effects (level 1 association between HRV and the EMA outcomes) and random slopes (the participants themselves were allowed to differ from each other in level 2). For each model, a control model was first created using only TST and RHR, followed by the full model that also included HRV. The marginal and conditional R^2^ of each model were then computed, which respectively represent the proportion of the variance that can be explained solely by the fixed effects (HRV, TST and RHR) and by the combination of the fixed and random effects (the participant). Differences in the marginal and conditional R^2^ between the control and full models were also calculated to assess (changes in) the goodness-of-fit of the models.

During statistical analysis, relatively large differences were found in the marginal and conditional R^2^ in each of the created models. To facilitate interpretation of these relatively large differences in the variance that was explained by the fixed and the combination of fixed random effects, three versions of the Coefficient of Variation (CV) of each variable were calculated to explore how the within-subject variance, the between-subject variance and the overall variance in the dataset compared to each other. The first version describes the average within-subject CV for each variable, and was determined by first calculating the within-subject CV based on the values of each participant (standard deviation divided by the mean) and then calculating the mean of those values. A CV of 0 was imputed for the (7) participants that had collected only one complete observation. The second version describes the between-subject CV for each variable, and was calculated by first determining the mean value for each participant and then calculating the CV of those values. The third version describes the overall CV for each variable, and consisted of the CV of the full dataset without accounting for within- or between-subject differences.

## Results

Of the 73 recruited participants, 63 collected at least one complete observation that included valid sleep, HRV and morning EMA data. The participation period per analyzed participant ranged from 1 to 57 days, with a median of 44 days. During these periods, the analyzed participants collected complete data on 1 to 46 days, with a median of 15 days. A total of 571 complete observations were analyzed. Due to training-related circumstances, the marines in training could temporarily not use their smartphones and thus collect data. The descriptive statistics for and intercorrelations between the independent (HRV, TST and RHR) and dependent (EMA) items of the analyzed dataset are presented in Table 1. A strong (r = .77; p < .001) correlation between perceived mental and physical fitness was found.

**Table 1:** Descriptive statistics for and intercorrelations between the daily measurements

Variable	Mean (SD)	Correlation			
		1	2	3	4
1. TST (hours)	6.22 (1.90)	–			
2. RHR (beats per minute)	61.80 (8.88)	− .09*	–		
3. lnrMSSD (milliseconds)	3.83 (0.40)	− .03	− 64***	–	
4. Perceived physical fitness (0–10)	7.84 (1.37)	.04	.03	.01	–
5. Perceived mental fitness (0–10)	8.11 (1.27)	.05	.10*	− .08●	.77***

*N* = *63*, *n* = *571*

*TST* total sleep time, *RHR* resting heart rate, *lnrMSSD* logarithmically transformed root mean square of the successive differences, a measure for heart rate variability (HRV)

***p < *.001*, **p < *.01*, *p < *.05*, ●*p* < *.1*

### Analysis 1: Perceived Physical Fitness

A two-step hierarchical linear mixed model for perceived physical fitness was created (Table 2). After controlling for TST and RHR, resting HRV during sleep was a statistically significant (p = .005) predictor of perceived physical fitness on the subsequent morning. Based on this finding, participants reported a higher perceived physical fitness on mornings after a sleep episode during which they also had a relatively high resting HRV. RHR significantly (p = .03) predicted perceived physical fitness in the control model (step 1), but not in the final model that also included HRV (step 2). Participants also tended (p = .10) to report a higher perceived physical fitness on mornings that followed a sleep episode with a relatively high TST. The explained variance of the fixed effects in the full model that included HRV (step 2) increased with 1.2% to a total of 3.1% in comparison to the control only model that was based on TST and RHR (step 1). The combination of the fixed and random effects explained 57.7% of the variance in the control model and 58.9% of the full model.

**Table 2:** Hierarchical linear mixed model for perceived physical fitness

Independent variable	Perceived physical fitness	
	Step 1	Step 2
	β	β
Intercept	− 0.053	− 0.087
TST	0.051	0.052●
RHR	− 0.101*	− 0.066
HRV		0.124*
*Marginal R*^*2*^	*0.013*	*0.031*
*∆ Marginal R*^*2*^		*0.018*
*Conditional R*^*2*^	*0.577*	*0.589*
*∆ Conditional R*^*2*^		*0.012*

N = 63, n = 571

*TST* total sleep time, *RHR* resting heart rate, *HRV* heart rate variability

*p < .05, ●*p* < .1

### Analysis 2: Perceived Mental Fitness

Another two-step hierarchical linear mixed model on perceived mental fitness was created (Table 3). After controlling for TST and RHR, resting HRV during sleep was not a statistically significant predictor of perceived mental fitness on the subsequent morning. TST was positively associated with perceived mental fitness (p = .04), as participants reported a higher perceived mental fitness on mornings that followed a sleep episode with a relatively high TST. Only 0.4% of the variance could be explained by the fixed effects in the full model, whereas 63.4% of the variance was explained by the combination of the fixed and random effects.

**Table 3:** Hierarchical linear mixed model for perceived mental fitness

Independent variable	Perceived mental fitness	
	Step 1	Step 2
	β	β
Intercept	− 0.052	− 0.059
TST	0.057*	0.058*
RHR	− 0.009	− 0.002
HRV		0.025
*Marginal R*^*2*^	*0.004*	*0.004*
*∆ Marginal R*^*2*^		*0.000*
*Conditional R*^*2*^	*0.633*	*0.634*
*∆ Conditional R*^*2*^		*0.001*

N = 63, n = 571

*TST* total sleep time, *RHR* resting heart rate, *HRV* heart rate variability

*p < .05

#### Within-Subject, Between-Subject and Overall Coefficients of Variation

**Fig. 1:**
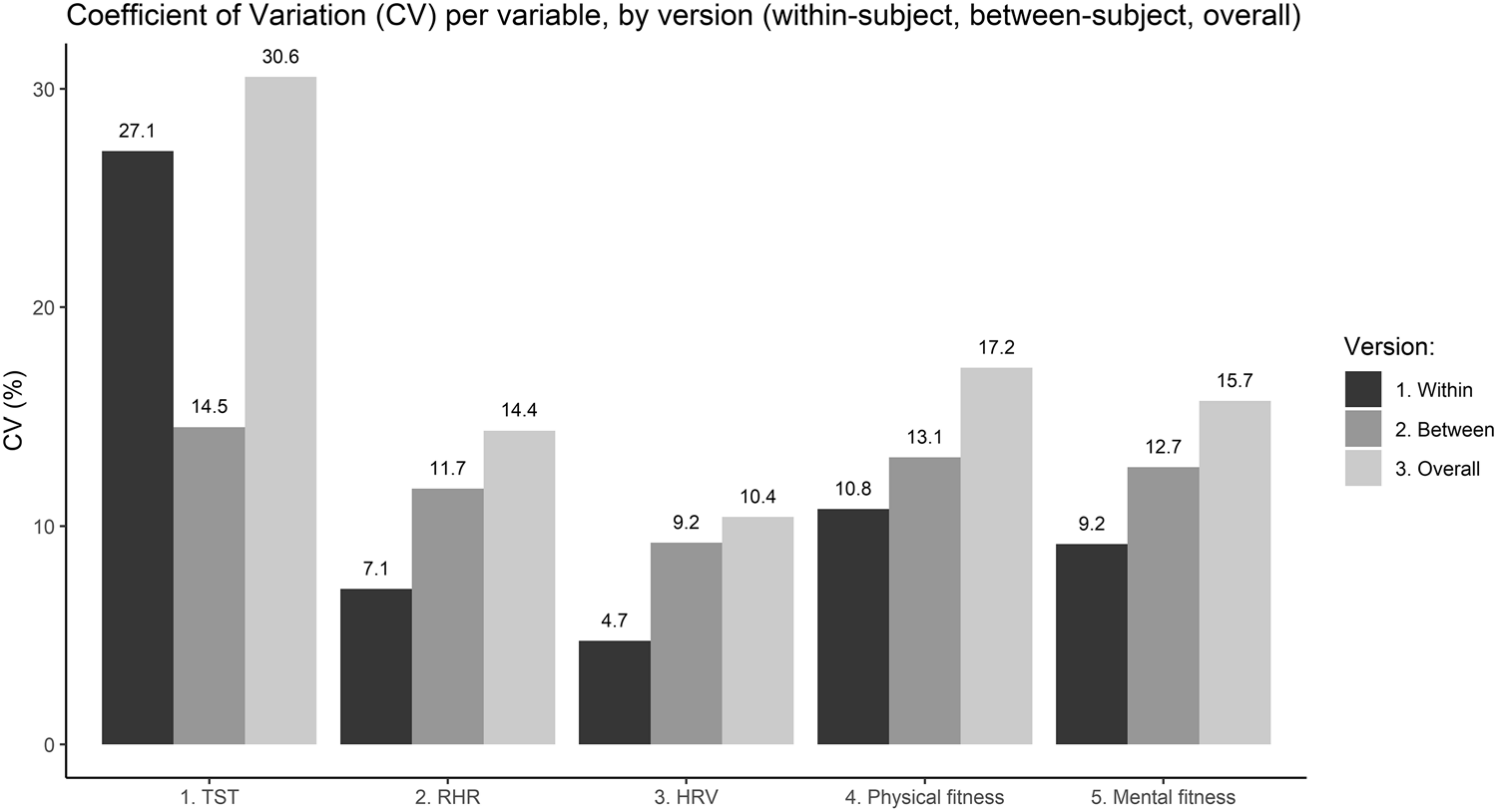
The coefficient of variation (CV) for the within-subject (left bar), between-subject (middle bar) and grand mean (right bar) version of each variable of the daily measurements

The within-subject, between-subject and overall CV for each predictor and outcome variable are visualized in Fig. 1. Two relevant observations can be made based on this data. First, participants reported consistently high scores on perceived mental and physical fitness (mean 7.84–8.11) with a limited tendency to also report low scores from time to time (SD 1.27–1.37). A second observation is that for perceived mental and physical fitness and particularly resting HRV, a relatively low amount of within-subject variance was available in the data in comparison to the between-subject and overall variance. This combination of findings indicates that there was a relatively modest amount of within-subject variance available for both outcome measures as well as the central predictor, which may have contributed to the relatively low explained variance of the fixed effects (marginal R^2^) in relation to the explained variance of the combination of the fixed and random effects (conditional R^2^).

## Discussion

This study aimed to explore to what extent wearable-measured resting heart rate variability (HRV) during sleep predicts the perceived mental and physical fitness of military personnel on the subsequent morning. After controlling for total sleep time (TST), resting HRV during sleep was a small but statistically significant predictor of perceived physical fitness, but not of perceived mental fitness. The current study yielded several insights that are relevant for future research on this topic. We will first provide a more in-depth interpretation of the findings and how they relate to prior research, then address strengths and limitations of this study, and finally provide recommendations for practice and future research.

### Interpretation of the Results

Wearable-measured resting HRV during sleep was a statistically significant positive predictor of perceived physical fitness on the subsequent morning. Although no prior studies utilizing a within-subject design to assess these relationships were identified, these results are in line with prior research that showed that between-subject differences in resting HRV are positively associated with cardiovascular fitness (Souza et al., 2021; Tomes et al., 2020) and negatively associated with overuse injuries (Gisselman et al., 2016; Lima-Borges et al., 2018; Williams et al., 2017) and pain perception (Forte et al., 2022). However, resting HRV explained only a small portion of the variance in perceived physical fitness (3.1% after controlling for TST and RHR).

Unlike hypothesized, wearable-measured resting HRV during sleep did not predict perceived mental fitness on the subsequent morning. To our knowledge, no prior studies have assessed the direct association between within-subject differences in resting HRV during sleep and perceived mental fitness on the subsequent morning, but between-subject differences in resting HRV have been positively associated with emotion regulation (Holzman & Bridgett, 2017; Mather & Thayer, 2018) and resilience (An et al., 2020; Hourani et al., 2020). However, within-subject differences in resting HRV were recently found to buffer against the associations between stress and negative affect (da Estrela et al., 2021), as well as between demands and stress or stress and mental exhaustion (de Vries et al., 2021). It is therefore possible that despite not being directly associated to perceived mental fitness in the current study, resting HRV could play a relevant role as a (psycho)physiological resource during appraisal and/or emotion regulation regardless.

In the current study, perceived mental and physical fitness were assessed via two items in a short EMA questionnaire and therefore represent the participant’s subjectively experienced mental and physical fitness rather than the underlying objective capacities. This, combined with the finding that the items on perceived mental and physical fitness were strongly correlated (r = .77), means that the found association between resting HRV and perceived physical fitness is reflective of a psychological state. Since psychological states can influence the perception of bodily sensations such as pain and vice versa (Loggia et al., 2008), the potential influence of the items on perceived mental and physical fitness may be bidirectional. Although both items can therefore be seen as different components of the perceived overall fitness that is assessed as a psychophysiological resource during appraisal, the current results suggest that resting HRV during sleep may be more related to the physical component of perceived fitness rather than the perceived mental component.

The comparison of the CVs (Fig. 1) showed that there was a relatively low amount of within-subject variance in the two perceived fitness measures as well as the central predictor HRV in comparison to the between-subject and overall variance. Several possible explanations for this can be given. For instance, the participants collected data during a relatively short period (1–57 days: median 44 days). As a result, there were a relatively modest number of complete observations per participant that could be analyzed (1–46 observations: median 15 observations). Since a lack of relevant variance (e.g., floor or ceiling effects) can contribute to false negative conclusions (Šimkovic & Träuble, 2019), it is possible that this may have contributed to a potential underestimation of the strength of the associations and thus the low explained within-subject variance (marginal R^2^).

Finally, the results showed that RHR had a negative correlation (r = -0.64; Table 1) with resting HRV and similar associations with mental and physical fitness. Neither RHR were related to mental fitness, but both RHR (Table 3, step 1) and resting HRV (Table 3, step 2) were linked to physical fitness. However, physical fitness was less strongly associated with RHR than with resting HRV, which was the only significant predictor in the full model where both were included. This observation aligns with that of a recent large-scale study which showed that RHR and resting HRV have similar associations to stress-related measures and concluded that resting HRV is a more sensitive but not specific marker of stress (Altini & Plews, 2021).

### Strengths and Limitations

A strength of this study is that it was based on data that was collected in a real-life setting, optimizing the generalizability of the findings. Furthermore, by utilizing an open-source sleep detection algorithm and a publicly available IBI artefact filtering method, the methods were transparent and reproducible. For instance, the used sleep detection algorithm and IBI artefact filtering method could in future research or applications be combined with hardware of another manufacturer.

Despite these advantages, a potential downside of using a novel open-source sleep detection algorithm is that it may be less accurate than algorithms of commercial wearable manufacturers that have more resources available for research and development. In the current study, the measurement of resting HRV during sleep directly depends on the respective sleep detection algorithm to ensure that the collected inter-beat-interval data is measured within the desired context. Potential inaccuracies in the sleep detection algorithm may therefore result in heart rate data of awake periods being included in the calculation of the resting HRV. Since motion artefacts are more likely to be present during awake periods, the accuracy of the HRV measurement may be indirectly affected by it. Potential inaccuracy in the detection of sleep and measurement of the related resting HRV may therefore have added error variance to the data, potentially leading to an underestimation of the strength of the associations that were tested. Another limitation of the current study was that a convenience sample was used where no data on the participants’ age, gender, function or reasons for missing data or drop-outs could be logged due to privacy and security concerns related to the profession of this military personnel. Since this study primarily focused on short-term, within-subject associations, this limitation did not impact the accuracy or relevance of the current results. However, as a result, no subgroup analyses could be performed to assess potential differences in the investigated associations among participants of different ages, gender of function groups. This also impacts the generalizability of the current findings, as it limits potential extrapolation to similar populations.

### Recommendations for Practice

This study presented relatively modest findings on associations between sleep, resting HRV and perceived mental and physical fitness. Although the found associations where relatively modest, the insights gained from this exploration using novel methods can be used to guide future use in future research and practice and thus provide a relevant contribution to the broader purpose of this body of knowledge; to eventually provide individuals with relevant and timely feedback on their readiness to handle demands and cope with stress. This segment will therefore first reflect on how the current findings should be interpreted for practice, whereas the next segment will describe more detailed recommendations for future studies.

Wearable-measured resting HRV during sleep was positively associated with perceived physical fitness in the current study, but explained only a small portion of its variance (3.1% after controlling for TST and RHR). Resting HRV during sleep should therefore not be seen as a potential replacement of perceived physical fitness, but as a complement to it. Prior studies showed that utilizing resting HRV measurements to guide training-related decision making can lead to positive outcomes in comparison to predefined training (Düking et al., 2021; Manresa-Rocamora et al., 2021). Therefore, resting HRV during sleep may be useful as a complement to the perceived physical fitness to guide decision-making on the physical readiness of the respective individual on the following day. Within this context, a resting HRV that is relatively high for the individual’s own standards can be seen as a favorable sign of physical fitness, whereas a low resting HRV would reflect the opposite.

Based on the current results, resting HRV during sleep does not appear to be directly associated to the perceived mental fitness. However, recent studies showed that waking up with a relatively favorable (within-subject) resting HRV appears to buffer against the negative impact of demands and stress (da Estrela et al., 2021; de Vries et al., 2021). It is therefore possible that resting HRV has no or a limited direct association to perceived mental fitness, but does function as a psychophysiological resource that allows the individual to flexibly adapt to challenges and thus as a component of the underlying mental fitness itself. Future research is needed to better understand the potential role of resting HRV in this process of resilience.

### Recommendations for Future Studies

Several recommendations for future studies on improving the accuracy of the sleep and related resting HRV measures, as well as how to assess the potential role of resting HRV as a measure of (perceived) fitness. The capacity of wearable technology to detect sleep affects the accuracy of the resting HRV measurements that are automatically collected within those periods. Three potentially promising approaches to measure resting HRV in a daily-life setting using consumer wearables can be considered by future studies. First, contributing to the development of open-source sleep detection algorithms and using more recent and optimized iterations of them will result in optimally transparent and reproducible methods (van Hees et al., 2018). Another approach for studies in which full custody of the collected data is required is to utilize the sleep algorithms of the used wearable devices itself and load the aggregated data of the full sleep episode directly from the wearable. For the present study, only accelerometer and inter-beat-interval data were available, but the latest versions of the Garmin Health SDK now also allow the extraction of the sleep data as classified by Garmin’s sleep algorithm (Garmin, 2022), of which the validity has been studied (Chinoy et al., 2020; Mouritzen et al., 2020; Stone et al., 2020). Finally, studies in domains with more lenient data storage requirements can also consider using consumer-available wearables that have been directly validated to accurately measure the resting HRV during sleep, such as the Oura ring (Cao et al., 2022; Kinnunen et al., 2020; Stone et al., 2021).

Besides optimizing the sleep and resting HRV measurement of wearables, future studies can consider taking a different approach in determining how HRV may be associated with (perceived) mental or physical fitness. Two recent studies showed that within-subject differences in resting HRV had a moderating effect on the associations between stress and negative affect (da Estrela et al., 2021), as well as on demands and stress and stress and mental exhaustion (de Vries et al., 2021). This is consistent with the neurovisceral integration model, which considers (vagally mediated) resting HRV itself to be an index of relatively optimal nervous system functioning to support adaptability to environmental demands (Thayer & Lane, 2000; Thayer et al., 2009). Therefore, it is possible that wearable-measured resting HRV is not (strongly) correlated with perceived physical or mental fitness as was found in this study, but does directly act as a psychophysiological resource during the processes of appraisal or emotion regulation and thus as a relevant but perhaps subconscious component of mental fitness. Future studies are therefore recommended to further explore this potentially direct role of resting HRV as a psychophysiological resource on fitness or similar resilience-related outcomes on a within-subject level. Furthermore, the present study and discussed recent studies primarily assess within-day associations of resting HRV. Although this approach is important to better understand the short-term relationship of differences in resting HRV with these outcomes, studies assessing longitudinal relationships are also needed to explore the potential impact of within-subject trends in resting HRV on a larger timeframe.

Finally, future research could further explore the mechanisms that were proposed in this article. For instance, by assessing how perceived measures of mental and physical fitness relate to objective observations of fitness, as well as general health and functioning, and if it can be improved through training. Although the short EMA-questionnaires that were used in this study are likely preferable for longer and more intensive (daily) data collection, future studies with a different design could also consider using more detailed questionnaires, for instance (a subscale of) the recently introduced and validated Acute Readiness Monitoring Scale that also specifically differentiates between mental and physical readiness (Keegan et al., 2021). Future studies in target populations with less privacy-related limitations should also include the analysis of whether the strength of these associations differs between individuals, for instance based on personal characteristics (e.g., age, gender, function-group).

## Supplementary Information

Below is the link to the electronic supplementary material.

Supplementary file1 (DOCX 51 KB)

## Data Availability

Data of this article are not publicly available due to the personal nature of the data in combination with the sensitive profession of the participants of this study.

## Footnotes

1 Upon request during peer-review, the Standard Deviation of the NN intervals (SDNN) time domain HRV metric was also calculated and analyzed as an alternative to the rMSSD. SDNN during sleep was not found to be significantly associated with morning mental and physical fitness. For transparency, the findings of these additional alternative analyses are available in Online Appendix 1.
