# Supplementary material for: Does Wearable-Measured Heart Rate Variability During Sleep Predict Perceived Morning Mental and Physical Fitness?
Source: Appl Psychophysiol Biofeedback. 2023 Jan 9;48(2):247–57. doi: 10.1007/s10484-022-09578-8 (PMC10195711; doi:10.1007/s10484-022-09578-8)
Supplement: Supplementary file 1 — Supplementary file1 (DOCX 51 KB) [file 10484_2022_9578_MOESM1_ESM.docx]

# Online appendix 1

| **Table 1**: Hierarchical linear mixed model for perceived physical fitness | | | | |
| --- | --- | --- | --- | --- |
|  | **Perceived physical fitness** | | | |
|  | **Step 1** | | **Step 2** | |
| **Independent variable** | **β** | | **β** | |
| Intercept | -0.053 |  | -0.070 |  |
| TST | 0.051 |  | 0.039 |  |
| RHR | -0.101 | * | -0.105 | * |
| SDNN |  |  | 0.066 |  |
|  |  |  |  |  |
| *Marginal R^2^* | *0.013* |  | *0.023* |  |
| *Δ Marginal R^2^* |  |  | *0.019* |  |
| *Conditional R^2^* | *0.577* |  | *0.580* |  |
| *Δ Conditional R^2^* |  |  | *0.011* |  |
| *Note. N=63, n=571; * p<.05; TST: Total Sleep Time; RHR: Resting Heart Rate; SDNN: Standard Deviation of the NN intervals, a measure for Heart Rate Variability (HRV)* | | | | |

| **Table 2**: Hierarchical linear mixed model for perceived mental fitness | | | | |
| --- | --- | --- | --- | --- |
|  | **Perceived mental fitness** | | | |
|  | **Step 1** | | **Step 2** | |
| **Independent variable** | **β** | | **β** | |
| Intercept | -0.052 |  | -0.061 |  |
| TST | 0.057 | * | 0.051 | . |
| RHR | -0.009 |  | -0.012 |  |
| SDNN |  |  | 0.035 |  |
|  |  |  |  |  |
| *Marginal R^2^* | *0.004* |  | *0.005* |  |
| *Δ Marginal R^2^* |  |  | *0.001* |  |
| *Conditional R^2^* | *0.633* |  | *0.632* |  |
| *Δ Conditional R^2^* |  |  | *-0.001* |  |
| *Note. N=63, n=571; * p<.05, . p<.1; TST: Total Sleep Time; RHR: Resting Heart Rate; SDNN: Standard Deviation of the NN intervals, a measure for Heart Rate Variability (HRV)* | | | | |
